# Metabolic syndrome in indigenous communities in Mexico: a descriptive and cross-sectional study

**DOI:** 10.1186/s12889-020-8378-5

**Published:** 2020-03-17

**Authors:** Elvia Cristina Mendoza-Caamal, Francisco Barajas-Olmos, Humberto García-Ortiz, Isabel Cicerón-Arellano, Angélica Martínez-Hernández, Emilio J. Córdova, Marcelino Esparza-Aguilar, Cecilia Contreras-Cubas, Federico Centeno-Cruz, Miguel Cid-Soto, Mirna Edith Morales-Marín, Adriana Reséndiz-Rodríguez, Juan Luis Jiménez-Ruiz, María Guadalupe Salas-Martínez, Yolanda Saldaña-Alvarez, Elaheh Mirzaeicheshmeh, María Rosalba Rojas-Martínez, Lorena Orozco

**Affiliations:** 1 Clinical Area, Instituto Nacional de Medicina Genómica, SS, Periférico Sur 4809, Colonia Arenal Tepepan, Delegación Tlalpan, C.P. 14610 Ciudad de México, Mexico; 2 Immunogenomics and Metabolic Disease Laboratory, Instituto Nacional de Medicina Genómica, SS, Periférico Sur 4809, Colonia Arenal Tepepan, Delegación Tlalpan, C.P. 14610 Ciudad de México, Mexico; 3 Epidemiology Research Department, Instituto Nacional de Pediatría, Insurgentes Sur 3700, Letra C, Colonia Insurgentes Cuicuilco, Delegación Coyoacán, C.P. 04530 Ciudad de México, Mexico; 4 Public Health Research Center, Instituto Nacional de Salud Pública, 7a Cerrada de Fray Pedro de Gante 50, Colonia Sección XVI, Delegación Tlalpan, C.P. 14080 Ciudad de México, Mexico

**Keywords:** Mexican Amerindian, Indigenous, Metabolic syndrome, Prevalence, HDL-cholesterol, Waist circumference, Triglyceride, Blood pressure, Fasting glucose

## Abstract

**Background:**

An Amerindian genetic background could play an important role in susceptibility to metabolic diseases, which have alarmingly increased in recent decades. Mexico has one of the highest prevalences of metabolic disease worldwide. The purpose of this study was to determine the prevalence of metabolic syndrome and its components in a population with high Amerindian ancestry.

**Methods:**

We performed a descriptive, quantitative, and analytical cross-sectional study of 2596 adult indigenous volunteers from 60 different ethnic groups. Metabolic syndrome and its components were evaluated using the American Heart Association/National Heart, Lung, and Blood Institute Scientific Statement criteria.

**Results:**

The overall prevalence of metabolic syndrome in the indigenous Mexican population was 50.3%. Although females had a higher prevalence than males (55.6% vs. 38.2%), the males presented with combinations of metabolic syndrome components that confer a higher risk of cardiovascular disease. The most frequent metabolic syndrome component in both genders was low HDL-cholesterol levels (75.8%). Central obesity was the second most frequent component in females (61%), though it had a low prevalence in males (16.5%). The overall prevalence of elevated blood pressure was 42.7% and was higher in males than females (48.8 vs. 40%). We found no gender differences in the overall prevalence of elevated triglycerides (56.7%) or fasting glucose (27.9%).

**Conclusions:**

We documented that individuals with Amerindian ancestry have a high prevalence of metabolic syndrome. Health policies are needed to control the development of metabolic disorders in a population with high genetic risk.

## Background

Metabolic syndrome (MetS) is a cluster of metabolic abnormalities that increase the risk of developing cardiovascular disease (CVD) [1], which has been the main cause of mortality over the last 20 years [2]. The prevalence of MetS varies widely across populations, and Latin-American and African populations have been reported to have greater susceptibility for the development of metabolic disorders [3–5]. Mexico in particular has had an alarming increase in recent decades, and currently has among the highest MetS prevalences worldwide [6]. Some studies have suggested that an Amerindian genetic background plays an important role in susceptibility to these diseases [4, 7–11], and that individuals with an Amerindian component may have genetic variants derived from natural selection that could be contributing factors in this epidemic, in addition to lifestyle changes [7, 8, 12–14]. The vast majority of the Mexican population is Mestizo with a strong Amerindian component (56%) [15], and an estimated 14.9% of the Mexican population comprises indigenous people distributed over 68 ethnic groups [16, 17]. Although many of these indigenous populations have retained their genetic backgrounds, native languages, and socioeconomic structures over the centuries [16, 18], over the last few decades they have experienced dramatic changes in their lifestyles, primarily with regard to excessive energy intake and a sedentary lifestyle [19, 20]. Parallel with this progressive change, an increase in the prevalence of metabolic diseases has been observed [21–28].

To gain a deeper understanding of the progressive increase of the MetS prevalence and its determinants in Mexican people, we evaluated 2596 individuals from 60 ethnic groups with a high Amerindian ancestry.

## Methods

The study was conducted in accordance with the Declaration of Helsinki and was approved by the Research, Ethics, and Biosafety Human Committees of the Instituto Nacional de Medicina Genómica (INMEGEN) in Mexico City. This research was performed from November 2012 to October 2017 in agreement with the indigenous leaders and with the support of the National Commission for the Development of Indigenous Communities (CDI, from the Spanish “Comisión Nacional para el Desarrollo de Pueblos Indígenas”). All participants provided written informed consent, and authorities or community leaders participated as translators as necessary.

A descriptive, quantitative, and analytical cross-sectional study was carried out with a total of 2596 Mexican Amerindians (MAs) belonging to the Metabolic Analysis in an Indigenous Sample (MAIS) cohort. The MAIS cohort was recruited from 73 indigenous communities from 60 different ethnic groups. Because indigenous people are considered a vulnerable population, all community members were invited to participate through posters, presentations, and announcements on local radio. In this study, only ≥18-year-old MAs with complete anthropometric and biochemical data were included.

Individuals were considered MAs only if they identified themselves as indigenous, had parents and grandparents who were born in the same community, and spoke the native language. The ancestry was confirmed in a random sample of 1304 MAs using the 6.0 SNP array (Affymetrix) or GoldenGate genotyping assay (Illumina) containing 96 ancestry markers validated in other studies [29]. They had an average Amerindian ancestry of 95% (standard deviation 5.7%) [30]. Anthropometric, demographic, personal and family medical history, and lifestyle data were obtained through participant interviews, which were conducted by trained staff from the INMEGEN using a standardized questionnaire.

Anthropometric measurements and blood pressure (BP) were obtained as follows. Height, weight, and body composition were measured without shoes and minimal clothing using an electronic stadiometer (ADE Germany) and Body Composition Monitor (HBF-500 INT, OMRON). Waist circumference (WC) was measured midway between the inferior margin of the ribs and the border of the iliac crest using a flexible clinical measuring tape. BP was obtained using a digital blood pressure monitor (HEM-907XL, OMRON) following international recommendations. A peripheral blood sample was extracted after a fast of at least 8 h. Biochemical analyses, including fasting glucose (FG), total cholesterol (TC), high density lipoprotein cholesterol (HDL-C), and triglycerides (TGs), were determined in most communities using a Cholestech LDX Analyzer, whereas for individuals living near Mexico City, these analyses were performed using the Synchron CX5 Analyzer System (Beckman Coulter Fullerton, CA, USA). Ten percent of the samples were analyzed using both systems, demonstrating high reproducibility.

MetS and its components were evaluated according to the American Heart Association/National Heart, Lung, and Blood Institute Scientific Statement (AHA/NHLBI) criteria [1]. The diagnosis of MetS was established by the presence of three or more of the following five traits: abdominal obesity (WC ≥102 cm in males and ≥ 88 cm in females), hypertriglyceridemia (TGs ≥150 mg/dl), low HDL-C (< 40 mg/dl in males and < 50 mg/dl in females), elevated BP (systolic BP ≥130 mmHg or diastolic BP ≥85 mmHg, or previous diagnosis of hypertension), and elevated FG (≥100 mg/dl or previous diagnosis of type 2 diabetes). In this study, alcohol consumption and smoking was considered positive when individuals self-reported its habitual consumption.

### Statistical analysis

Results are reported as means ± standard deviation, or as percentages and 95% exact confidence intervals (CIs). We categorized participants into six age groups and stratified by gender to facilitate comparison. Metabolite levels were compared between genders using the Student’s t-test and prevalence rates using the chi-squared test. Logistic regression analyses were performed to predict potential significant predictor of MetS (body mass index (BMI), visceral fat, corporal fat, alcohol consumption, smoking, and family records of type 2 diabetes and hypertension). Data were analyzed using R, version 3.1.0. *P*-values < 0.05 were considered significant.

## Results

MAIS cohort had 3021 adult Mexican volunteers belonging to 60 ethnic groups. A total of 2596 adult MAs, who met the inclusion criteria were enrolled in this study (Table 1). We observed a significant differential distribution between both genders in 12 of the 14 metabolic and anthropometric parameters evaluated. We found that mean WC, TGs, FG, systolic and diastolic BP, visceral fat percentage, and hypertension, alcoholic consumption and smoking prevalences were higher among males, who also had a lower mean HDL-C. Mean BMI and body fat percentages were higher among females.

**Table 1:** Baseline Characteristics of Study Participants by Gender

Characteristic	Overall	Females	Males	*P*-value^*^
Participants, n (%)	2596 (100%)	1809 (70%)	787 (30%)	
Age, years	47.9 ± 16.4	52.4 ± 16.8	45.9 ± 15.8	< 0.0001
Waist circumference, cm	91.4 ± 10.9	91.0 ± 11.2	92.0 ± 10.3	0.03
Triglycerides, mg/dL	189.0 ± 108.6	185.9 ± 104.3	196.3 ± 117.8	0.003
HDL-C, mg/dL	39.5 ± 12.1	40.1 ± 12.9	37.9 ± 12.6	< 0.0001
Glucose, mg/dL	102.9 ± 50.6	102.5 ± 51.8	103.7 ± 47.6	< 0.0001
Systolic blood pressure, mmHg	126.0 ± 22.5	124.3 ± 22.3	129.9 ± 22.3	< 0.0001
Diastolic blood pressure, mmHg	73.2 ± 12.0	72.0 ± 11.6	75.0 ± 12.6	< 0.0001
Total cholesterol, mg/dL	177.4 ± 39.4	177.31 ± 39.4	177.5 ± 39.6	0.949
Body mass index, kg/m^2^	27.5 ± 4.9	27.9 ± 5	26.4 ± 4.2	< 0.0001
Visceral fat, %	9.1 ± 3.8	8.3 ± 3.1	11 ± 4.7	< 0.0001
Body fat, %	36.3 ± 10.6	41.3 ± 7.5	24.9 ± 7.5	< 0.0001
Type 2 diabetes (PD), %	13.7 (12.4–15.1)	14.3 (12.7–16)	12.3 (10.1–14.8)	0.186
Hypertension (PD), %	13.6 (12.3–15)	11.8 (10.4–13.4)	17.8 (15.2–20.6)	< 0.0001
FDR with diabetes, %	21.5 (20–23.2)	21.9 (20.1–23.9)	20.6 (17.8–23.6)	0.597
FDR with hypertension, %	26.9 (25.2–28.7)	26.6 (24.6–28.7)	27.7 (24.6–31)	0.729
Alcohol consumption, %	13 (11.9–14.2)	2 (1.5–2.7)	33.4 (30.6–36.3)	< 0.0001
Smoking, %	7.9 (7–8.8)	1.7 (1.2–2.3)	20.1 (17.8–22.6)	< 0.0001

Data are presented as mean ± standard deviation or frequency (95% confidence intervals) unless otherwise noted. PD, previous diagnostic; FDR, first-degree relationship.

*Comparison of females and males

**Fig. 1:**
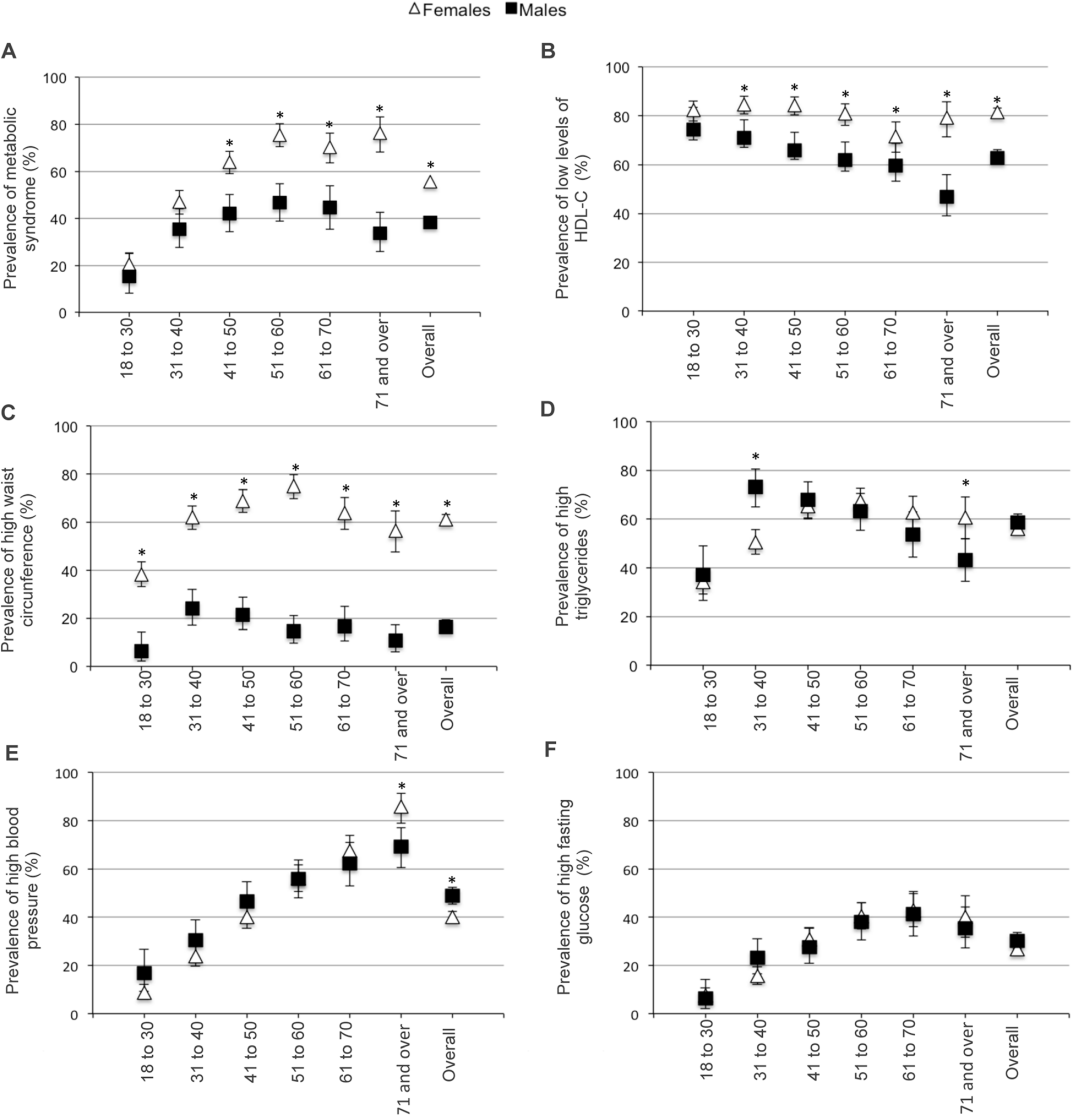
Prevalence of metabolic syndrome and its components by gender and age group. Calculated according to American Heart Association/National Heart, Lung, and Blood Institute Scientific Statement criteria. 95% confidence intervals are given. *P < 0.05 when comparing females and males

The overall prevalence of MetS in this population was 50.3% (95% CI 48.4–52.3%). The prevalence of MetS and its components stratified by age group and gender are shown in Fig. 1 and Additional file 1. Females had a significantly higher prevalence of MetS than males (55.6 vs. 38.2%, *P* < 0.0001; Fig. 1a). Interestingly, we found no significant difference between groups ≤40 years old, whereas females in age groups > 40 years had 1.5 to 2.3-times higher MetS prevalence than males.

Detailed MetS analysis showed that the most frequent component for both genders was low HDL-C levels (75.8; 95% CI 74.2–77.5%), with a significantly higher frequency in females relative to males regardless of age (81.5 vs. 62.8%, *P* < 0.0001), except in the youngest age group, in which there was no difference between genders (Fig. 1b).

Females had a significantly higher frequency of central obesity than males across all age groups (61 vs. 16.5%, *P* < 0.0001; Fig. 1c), and elevated WC was the second most frequent component of MetS in females. We observed an overall prevalence of hypertriglyceridemia of 56.7% (95% CI 54.8–58.6%), with no difference between genders (females: 55.9% vs. males: 58.6%; Fig. 1d). However, when we stratified by age, significant differences were found between males and females. Specifically, males between 31 and 40 years of age had a higher frequency of hypertriglyceridemia than females in the same age group (73.2 vs. 50.6%, *P* < 0.0001), whereas the opposite pattern was observed in the ≥71 years age group, in which females had a higher prevalence (60.7 vs. 43.1%, *P =* 0.004).

The overall prevalence of elevated BP was 42.7% (95% CI 40.8–44.6%). This was higher in males (48.8 vs. 40%, *P* < 0.0001; Fig. 1e), though after stratifying by age and gender, the females in the last age group had a higher prevalence of elevated BP than males (85.9 vs. 69.2%, *P* = 0.002).

**Fig. 2:**
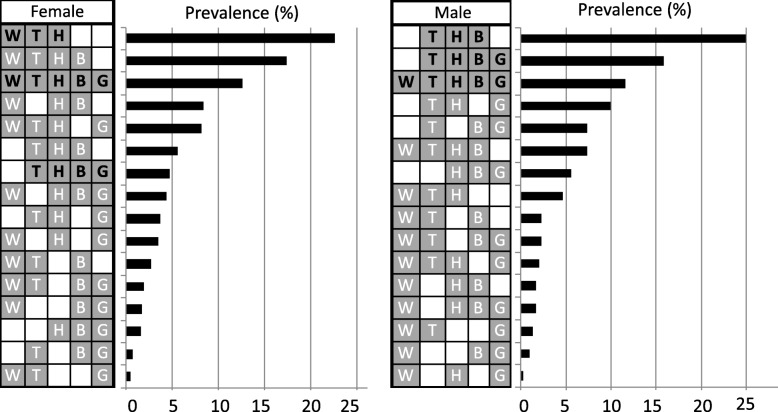
Prevalence of the 16 combination types according to gender. 95% confidence intervals (CIs) are given. H, HDL-C; W, waist circumference; T, triglycerides; B, blood pressure; G, fasting glucose

The overall prevalence of elevated FG was 27.9% (95% CI 26.1–29.6%), with a parallel increase with age and no difference between genders (Fig. 1f). Notably, we found large differences between males and females with regard to MetS component combinations (Fig. 2). The most frequent combination observed in females was central obesity, hypertriglyceridemia, and low HDL-C levels, whereas in males it was elevated BP, hypertriglyceridemia, and low HDL-C levels. The combination that includes the presence of all five components ranked third in both genders. Otherwise, we found that the obesity (OR 4.5; 95% CI 3.7–5.6), a high percentage of body fat (OR 4.6; 95% CI 3.8–5.6) or visceral fat (OR 3.2; 95% CI 2.2–4.8) and a history of two parents with diabetes (OR 3.2; 95% CI 1.8–5.8) conferred the greatest risk for MetS development in an independent manner (Table 2).

**Table 2:** Adjusted Odd Ratios (ORs) for Metabolic Syndrome

Variable		OR (95% CI)	Adjusted OR^*^ (95% CI)
Total cholesterol	≥ 200 mg/dl or medical treatment	2.2 (1.8–2.6)^†^	1.9 (1.6–2.4)^b^
Obesity	BMI ≥ 30 kg/m^2^	4.1 (3.35–4.9)^†^	4.5 (3.7–5.6)^b^
Body fat, %	High^a^	3.2 (2.7–3.8)^†^	4.6 (3.8–5.6)^b^
Visceral fat	High (≥10%)	2.4 (1.6–3.5)^†^	3.2 (2.2–4.8)^b^
First-degree	Mother	1.6 (1.2–1.9)^†^	1.8 (1.4–2.3)^b^
relationship	Father	1.6 (1.2–2.2)^†^	1.9 (1.4–2.7)^b^
with type 2 diabetes	Both parents	2.8 (1.6–5.0)^†^	3.2 (1.8–5.8)^b^
First-degree	Mother	1.4 (1.1–1.7)^†^	1.6 (1.3–2.0)^b^
relationship	Father	1.4 (1.0–1.8)^†^	1.4 (1.0–1.9)
with hypertension	Both parents	1.6 (1.0–2.6)	1.6 (1.0–2.6)
Alcohol consumption	True	0.7 (0.6–1.0)	1.3 (0.9–1. 7)
Smoking	True	0.9 (0.7–1.3)	1.6 (1.2–2.2)^c^

*Adjusted by age and gender

^a^ Cutoff value [31]

^b^*P* < 0.0001

^c^*P* = 0.01

## Discussion

In this study, we found that the prevalence of MetS in MAs is 50.3% according to AHA/NHLBI. Thus, MAs have one of the highest prevalences of MetS worldwide, even higher than that reported in the Mexican Mestizo population (41.6%) by the National Health Survey of Mexico (ENSANUT) [6], and twice that reported for the Latin-American population (24.9%) [5].

Before the 1980s, indigenous people were suggested to have protective genetic factors against metabolic diseases, as these populations had a low prevalence of metabolic disorders, such as type 2 diabetes and obesity [21, 22]. However, an acculturation of indigenous people started in Mexico after that decade, with a parallel increased in the prevalence of these diseases [21–28]. It was recently proposed that populations with an Amerindian origin may have selected for specific variants in genes that facilitated survival during periods of famine. Natural selection could be responsible for more efficient energy storage, which results in a high prevalence of metabolic disorders [12, 13]. Such significant changes in the prevalence of MetS could reflect the acculturation of a genetically susceptible population and the adoption of an obesogenic environment [19, 20, 24]. This assertion is supported by the observation that indigenous communities close to urban areas have a higher prevalence of metabolic disorders relative to communities with the same genetic background that remain isolated [19, 24, 32, 33, 34]. Examples of the genetic variants that could have been selected and that have the highest frequency in mestizo and indigenous populations are a haplotype spanning *SLC16A11* and conferring a higher risk of developing type 2 diabetes, and the *ABCA1*/R230C functional variant, which has been consistently associated with low HDL-C levels [7, 35]. The latter is the most prevalent MetS component in this study. Other genomic variants of Amerindian origin harbored by Mestizo population have been identified in various genes, such as *SIK3*, *RORA*, *LPL*, *APOA5*, *ANGPL3,* and *TIMD4*, which are involved in lipid metabolism and have been strongly associated with hypertriglyceridemia, suggesting that populations with an Amerindian background also have a greater genetic susceptibility to this entity [8, 14, 36]. In according with this, it has been previously reported that Mestizos have one of the highest prevalence of hypertriglyceridemia worldwide [8, 14, 36], and we observed that it was even higher in MAs (53.7 vs. 33%) [6]. A limitation of this study could be the sampling strategy, since being a vulnerable population the community was openly invited to participate. However, it is important point out that except to hypertriglyceridemia, the prevalence of the other MetS components was similar to that reported by ENSANUT, which used a probabilistic, multistage and stratified cluster sampling design [6], suggesting that our data reflect the current condition of MetS in the MA population.

With regard to the combinations of MetS components, we found significant differences between males and females. Females presented more frequently with the combinations that involved central obesity, whereas the males had combinations that included elevated BP. Previously published studies have shown that the risk of developing CVD is different according to the combination of MetS components. One of the combinations with higher risk of CVD is low HDL-C level, high BP, and elevated plasma TGs and FG [37, 38]. In this study, this combination was 3-fold more frequent in males than females (15.9 vs. 4.7%) and ranked second in frequency in males.

On the other hand, MetS prevalence also significantly different between males and females. However, after stratifying by age groups, we observed that the prevalence of MetS was similar in both genders for age ≤ 40 years old, whereas the oldest age group had a 2.3-fold higher prevalence in females than males. Notably, MetS prevalence did not increase in parallel with age in males. It is possible that the lower MetS prevalence observed in older males reflects lifestyle differences from females, specifically in relation to outdoor activities. However, we cannot discard the possibility that the observed difference is a reflection of lower survival in males, as national statistics indicate that a higher percentage of males die from CVD [39]. This hypothesis has several possible explanations. First, as mentioned previously, males present with combinations of MetS components that confer a higher risk for the development of CVD than females [37, 38]. Second, average systolic and diastolic BP and the prevalence of hypertension were higher in males than females, suggesting that the severity of this trait could be higher in males. Third, despite the fact that males had a lower prevalence of abdominal obesity, they had a higher percentage of visceral fat, which has been strongly associated with increased cardiometabolic risk [40].

Otherwise, as previously reported [41–43], obesity, body and visceral fat were the major determinants of MetS development, conferring a 4.5, 4.6 and 3.2-fold risk, respectively. Smoking just was revealed as a risk determinant to MetS after adjusted by age and gender. It is possible that this could has been influenced by its low prevalence observed in women. Acculturation has been associated with the smoking likelihood [44], but interestingly, we observed that in indigenous women this phenomenon seems to be limited to dietary habits and sedentary lifestyle.

Additionally, a family history of both parents with type 2 diabetes had a 3.2-fold risk of developing MetS. Therefore, genetic risk factors in indigenous people need to be studied to deepen the understanding of the pathophysiology of metabolic disorders in a highly susceptible population.

## Conclusions

Mexico has made multiple efforts to preserve the culture of their indigenous populations, but globalization and lifestyle changes have exposed this population at an alarming rate of acculturation. Health policies aimed at prevention and early detection are needed to control the increase in metabolic diseases in a population with, perhaps, a higher genetic risk for metabolic disorders.

## Supplementary information

**Additional file 1.** Prevalence of Metabolic Syndrome and its components by gender and age group. Description of data: Data are presented as frequency (95% confidence intervals) and prevalences were calculated according to American Heart Association/National Heart, Lung, and Blood Institute Scientific Statement criteria.

## Data Availability

The datasets generated and analysed during the current study are not publicly available due this work is part of a larger project but are available from the corresponding author on reasonable request.

## Abbreviations

AHA/NHLBI: American Heart Association/National Heart, Lung, and Blood Institute Scientific Statement
BMI: Body mass index
BP: Blood pressure
CDI: Comisión Nacional para el Desarrollo de Pueblos Indígenas
CI: Confidence interval
CVD: Cardiovascular disease
FG: Fasting glucose
HDL-C: High density lipoprotein cholesterol
INMEGEN: Instituto Nacional de Medicina Genómica
MA: Mexican Amerindian
MAIS: Metabolic Analysis in an Indigenous Sample
MetS: Metabolic syndrome
OR: Odds ratio
TC: Total cholesterol
TG: Triglyceride
WC: Waist circumference
