# Supplementary material for: Metabolic syndrome in indigenous communities in Mexico: a descriptive and cross-sectional study
Source: BMC Public Health. 2020 Mar 17;20:339. doi: 10.1186/s12889-020-8378-5 (PMC7076922; doi:10.1186/s12889-020-8378-5)
Supplement: Supplementary file 1 — Additional file 1. Prevalence of Metabolic Syndrome and its components by gender and age group. Description of data: Data are presented as frequency (95% confidence intervals) and prevalences were calculated according to American Heart Association/National Heart, Lung, and Blood Institute Scientific Statement criteria. [file 12889_2020_8378_MOESM1_ESM.doc]

**Additional file 1. Prevalence of Metabolic Syndrome and its components by gender and age group.**

|  | **Age group** | **Overall** | **Females** | **Males** | **P value*** |
| --- | --- | --- | --- | --- | --- |
|  | 18 to 30 years | 19.6 (15.9-23.7) | 20.6 (16.4-25.2) | 15.4 (8.2-25.3) | 0.359 |
|  | 31 to 40 years | 43.9 (39.7-48.3) | 46.9 (41.9-51.9) | 35.5 (27.6-44.1) | 0.045 |
| **Metabolic syndrome** | 41 to 50 years | 57.7 (53.4-61.8) | 63.9 (58.9-68.6) | 42.1 (34.4-50.2) | <0.0001 |
|  | 51 to 60 years | 65.8 (61.3-70) | 75.5 (70.5-80.2) | 46.6 (38.8-54.6) | <0.0001 |
|  | 61 to 70 years | 61.1 (55.6-66.3) | 70.2 (63.6-76.3) | 44.5 (35.4-53.9) | <0.0001 |
|  | 71 years and over | 55.5 (49.3-61.6) | 76.3 (68.2-83.2) | 33.8 (25.8-42.7) | <0.0001 |
|  | Overall | 50.3 (48.4-52.3) | 55.6 (53.3-57.9) | 38.2 (34.8-41.7) | <0.0001 |
|  | 18 to 30 years | 80.9 (76.8-84.5) | 82.3 (77.9-86.2) | 74.4 (63.2-83.6) | 0.106 |
| **Reduced HDL-** | 31 to 40 years | 81.2 (77.6-84.4) | 84.7 (80.8-88.1) | 71 (62.7-78.4) | 0.018 |
| **cholesterol** | 41 to 50 years | 79.1 (75.5-82.4) | 84.3 (80.4-87.8) | 66 (58.1-73.4) | <0.0001 |
|  | 51 to 60 years | 74.5 (70.3-78.3) | 80.9 (76.1-85) | 62 (54-69.4) | <0.0001 |
|  | 61 to 70 years | 67.4 (62-72.4) | 71.6 (65.1-77.5) | 59.7 (50.3-68.6) | 0.027 |
|  | 71 years and over | 63.4 (57.3-69.2) | 79.3 (71.4-85.8) | 46.9 (38.1-55.9) | <0.0001 |
|  | Overall | 75.8 (74.2-77.5) | 81.5 (79.7-83.3) | 62.8 (59.3-66.2) | <0.0001 |
|  | 18 to 30 years | 32.4 (27.9-37.1) | 38.3 (33.1-43.6) | 6.4 (2.1-14.3) | <0.0001 |
| **Elevated Waist** | 31 to 40 years | 52.1 (47.8-56.4) | 61.9 (56.9-66.7) | 23.9 (17.1-31.9) | <0.0001 |
| **Circumference** | 41 to 50 years | 55.3 (51.1-59.5) | 68.9 (64.1-73.5) | 21.4 (15.3-28.6) | <0.0001 |
|  | 51 to 60 years | 54.6 (50-59.1) | 74.9 (69.8-79.6) | 14.7 (9.7-21.1) | <0.0001 |
|  | 61 to 70 years | 47 (41.6-52.5) | 63.7 (56.9-70.2) | 16.8 (10.6-24.8) | <0.0001 |
|  | 71 years and over | 34 (28.3-40) | 56.3 (47.5-64.8) | 10.8 (6-17.4) | <0.0001 |
|  | Overall | 47.5 (45.6-49.5) | 61 (58.7-63.3) | 16.5 (14-19.3) | <0.0001 |
|  | 18 to 30 years | 34.8 (30.2-39.5) | 34.2 (29.2-39.5) | 37.2 (26.5-48.9) | 0.714 |
| **Elevated** | 31 to 40 years | 56.4 (52.1-60.7) | 50.6 (45.6-55.6) | 73.2 (65-80.4) | <0.0001 |
| **Triglycerides** | 41 to 50 years | 66.1 (62-70.1) | 65.4 (60.5-70.1) | 67.9 (60.1-75.1) | 0.599 |
|  | 51 to 60 years | 66 (61.6-70.2) | 67.4 (62-72.5) | 63.2 (55.3-70.6) | 0.382 |
|  | 61 to 70 years | 59.6 (54.1-64.9) | 62.8 (56-69.3) | 53.8 (44.4-63) | 0.131 |
|  | 71 years and over | 52.1 (45.9-58.2) | 60.7 (52-69) | 43.1 (34.4-52) | 0.005 |
|  | Overall | 56.7 (54.8-58.6) | 55.9 (53.6-58.2) | 58.6 (55-62) | 0.211 |
|  | 18 to 30 years | 10.2 (7.5-13.4) | 8.7 (5.9-12.2) | 16.7 (9.2-26.8) | 0.067 |
| **Elevated Blood** | 31 to 40 years | 25.5 (21.9-29.4) | 23.8 (19.7-28.3) | 30.4 (22.9-38.8) | 0.182 |
| **Pressure** | 41 to 50 years | 42 (37.8-46.2) | 40.2 (35.3-45.2) | 46.5 (38.6-54.6) | 0.200 |
|  | 51 to 60 years | 56 (51.5-60.5) | 56.1 (50.5-61.6) | 55.8 (47.9-63.6) | 1 |
|  | 61 to 70 years | 65.6 (60.2-70.7) | 67.4 (60.7-73.7) | 62.2 (52.8-70.9) | 0.516 |
|  | 71 years and over | 77.7 (72.2-82.6) | 85.9 (78.9-91.3) | 69.2 (60.5-77) | 0.002 |
|  | Overall | 42.7 (40.8-44.6) | 40 (37.8-42.3) | 48.8 (45.2-52.3) | <0.0001 |
|  | 18 to 30 years | 7.3 (5-10.2) | 7.5 (5-10.8) | 6.4 (2.1-14.3) | 0.912 |
| **Elevated Fasting** | 31 to 40 years | 17.5 (14.4-21) | 15.5 (12.1-19.5) | 23.2 (16.4-31.1) | 0.052 |
| **Glucose** | 41 to 50 years | 29.9 (26.1-33.9) | 30.8 (26.3-35.6) | 27.7 (20.9-35.3) | 0.548 |
|  | 51 to 60 years | 39.6 (35.2-44.1) | 40.4 (35-46) | 38 (30.6-46) | 0.660 |
|  | 61 to 70 years | 42.2 (36.9-47.7) | 42.8 (36.1-49.7) | 41.2 (32.2-50.6) | 0.914 |
|  | 71 years and over | 37.7 (31.9-43.9) | 40 (31.7-48.8) | 35.4 (27.2-44.2) | 0.486 |
|  | Overall | 27.9 (26.1-29.6) | 26.8 (24.8-28.9) | 30.2 (27-33.6) | 0.074 |

Data are presented as frequency (95% confidence intervals).

Prevalences calculated according to American Heart Association/National Heart, Lung, and Blood Institute Scientific Statement criteria.

*Comparison of females and males.
